# Human_SNP_TATAdb: a database of SNPs that statistically significantly change the affinity of the TATA-binding protein to human gene promoters: genome-wide analysis and use cases

**DOI:** 10.18699/VJGB-23-85

**Published:** 2023-12

**Authors:** S.V. Filonov, N.L. Podkolodnyy, O.A. Podkolodnaya, N.N. Tverdokhleb, P.M. Ponomarenko, D.A. Rasskazov, A.G. Bogomolov, M.P. Ponomarenko

**Affiliations:** Institute of Cytology and Genetics, Siberian Branch of the Russian Academy of Sciences, Novosibirsk, Russia Novosibirsk State University, Novosibirsk, Russia; Institute of Cytology and Genetics, Siberian Branch of the Russian Academy of Sciences, Novosibirsk, Russia Institute of Computational Mathematics and Mathematical Geophysics, Siberian Branch of the Russian Academy of Sciences, Novosibirsk, Russia; Institute of Cytology and Genetics, Siberian Branch of the Russian Academy of Sciences, Novosibirsk, Russia; Institute of Cytology and Genetics, Siberian Branch of the Russian Academy of Sciences, Novosibirsk, Russia; Institute of Cytology and Genetics, Siberian Branch of the Russian Academy of Sciences, Novosibirsk, Russia; Institute of Cytology and Genetics, Siberian Branch of the Russian Academy of Sciences, Novosibirsk, Russia; Institute of Cytology and Genetics, Siberian Branch of the Russian Academy of Sciences, Novosibirsk, Russia; Institute of Cytology and Genetics, Siberian Branch of the Russian Academy of Sciences, Novosibirsk, Russia

**Keywords:** TATA box, affinity, TBP, single nucleotide polymorphism, database, genome-wide analysis, ТАТА-бокс, аффинность, TBP, однонуклеотидный полиморфизм, база данных, полногеномный анализ

## Abstract

It was previously shown that the expression levels of human genes positively correlate with TBP affinity for the promoters of these genes. In turn, single nucleotide polymorphisms (SNPs) in human gene promoters can affect TBP affinity for DNA and, as a consequence, gene expression. The Institute of Cytology and Genetics SB RAS (ICG) has developed a method for predicting TBP affinity for gene promoters based on a three-step binding mecha- nism: (1) TBP slides along DNA, (2) TBP stops at the binding site, and (3) the TBP-promoter complex is fixed due to DNA helix bending. The method showed a high correlation of theoretical predictions with measured values during repeated experimental testing by independent groups of researchers. This model served as a base for other ICG web services, SNP_TATA_Z-tester and SNP_TATA_Comparator, which make a statistical assessment of the SNP-induced change in the affinity of TBP binding to the human gene promoter and help predict changes in expression that may be associated with a genetic predisposition to diseases or phenotypic features of the organism. In this work, we integrated into a single database information about SNPs in human gene promoters obtained by automatic extrac- tion from various heterogeneous data sources, as well as the estimates of TBP affinity for the promoter obtained using the three-step binding model and predicting their effect on gene expression for wild-type promoters and promoters with SNPs. We have shown that Human_SNP_TATAdb can be used for annotation and identification of candidate SNP markers of diseases. The results of a genome-wide data analysis are presented, including the distri- bution of genes with respect to the number of transcripts, the distribution of SNPs affecting TBP-DNA affinity with respect to positions within promoters, as well as patterns linking TBP affinity for the promoter, the specificity of the TBP binding site for the promoter and other characteristics of promoters. The results of the genome-wide analysis showed that the affinity of TBP for the promoter and the specificity of its binding site are statistically related to other characteristics of promoters important for the functional classification of promoters and the study of the features of differential gene expression.

## Abstract (ru)

Ранее было показано, что уровень экспрессии генов человека положительно коррелирует с аффинностью ТВР к промоторам этих генов. В свою очередь, однонуклеотидные полиморфизмы (SNP) в промоторах генов человека могут влиять на аффинность белка TBP к ДНК и, как следствие, на экспрессию генов. В ИЦиГ СО РАН разработан метод предсказания аффинности TBP к промоторам генов на основе трехшагового механизма связывания, включающего скольжение ТВР по ДНК, остановку ТВР в месте связывания, фиксацию комплекса ТВР–промотор за счет изгиба спирали ДНК. Метод показал высокую корреляцию теоретических предсказаний с измеренными значениями при многократной экспериментальной проверке независимыми группами исследователей. На основе этой модели в ИЦиГ СО РАН ранее были разработаны веб-сервисы SNP_TATA_Z-tester и SNP_TATA_Comparator, позволяющие вычислять статистическую оценку вызванного SNP изменения аффинности связывания TBP с промотором гена человека и прогнозировать изменение экспрессии, которые могут быть связаны с генетической предрасположенностью к заболеваниям или фенотипическими особенностями организма. В настоящей работе проведена интеграция в единой базе данных информации об однонуклеотидных полиморфизмах в промоторах генов человека, полученной путем автоматической экстракции из различных гетерогенных источников данных, а также результатов оценки аффинности TBP к промотору с использованием трехшаговой модели связывания и оценки их влияния на экспрессию генов для промоторов дикого типа и промоторов с однонуклеотидным полиморфизмом. Показана возможность использования базы данных Human_SNP_TATAdb для аннотации и выявления кандидатных SNP-маркеров заболеваний. Представлены результаты полногеномного анализа данных, включая особенности распределения генов по количеству транскриптов, распределение SNP, влияющих на аффинность TBP к ДНК по позициям внутри промоторов, а также закономерности, связывающие между собой аффинность TBP к промотору, специфичность сайта связывания TBP с промотором и другие характеристики промоторов. Результаты полногеномного анализа показали, что аффинность TBP к промотору и специфичность его сайта связывания статистически связаны с другими характеристиками промоторов, важными для функциональной классификации промоторов и исследования особенностей дифференциальной экспрессии генов.

## Introduction

The development of methods for predicting the effect of mutations on the level of gene expression for various organisms is important for solving many problems in the field of biotechnology, plant breeding, medicine, etc. Mutations in the human genome can be associated with a variety of physiological characteristics and diseases, and knowledge of their presence and cause is certainly necessary for the actively developing approach of personalized medicine.

The most common type of mutation in the human genome is SNP (Single Nucleotide Polymorphism), which is a single nucleotide difference in the DNA sequence. SNPs can be localized in different functional regions of the genome, which determines the nature of their influence. Mutations in the coding regions of the gene are the most studied; they directly affect the structure of the transcribed mRNA and, consequently, the synthesized protein. However, genome-wide association studies (GWAS) have shown that most SNPs that are significantly associated with disease susceptibility are located in non-coding regions (Hindorff et al., 2009; French and Edwards, 2020; Chandra et al., 2021), and more than 90 % of them are located in regulatory elements (Maurano et al., 2012). At the moment, one of the most studied regulatory regions is the TATA box region in the promoter, the sequence of which determines the affinity of the TBP protein (TATA binding protein), which is a key transcription initiation factor. Mutations in this region can affect the binding of the TBP protein to the promoter and, consequently, gene expression (Savinkova et al., 2007).

Previously, at the Institute of Cytology and Genetics SB RAS, a method for predicting the affinity of TBP for gene promoters based on a three-step binding mechanism was developed (Ponomarenko et al., 2008). The method showed a high correlation of theoretical predictions with experimentally measured affinity values when tested multiple times by independent groups of researchers (Delgadillo et al., 2009; Savinkova et al., 2013; Oshchepkov et al., 2022). Based on this model, the Institute of Cytology and Genetics SB RAS developed the SNP_TATA_Z-tester web service (Rasskazov et al., 2013), which allows one to calculate a statistical assessment of the SNP-induced change in the binding affinity of TBP for the human gene promoter and predict changes in expression. Using this web service, we previously identified candidate SNP markers for autoimmune diseases (Ponomarenko et al., 2016a), behavioral disorders (Chadaeva et al., 2016), chronopathologies (Ponomarenko et al., 2016b) and other diseases.

In this work, we integrated into a database information about SNPs in human gene promoters, obtained by automatic extraction from various heterogeneous data sources, as well as the results of assessing the affinity of TBP for the promoter and the specificity of the TBP binding site using a three-step binding model and assessing their effect on gene expression for the reference genome promoters and promoters with SNPs.

The main use of the Human_SNP_TATAdb database is the annotation of promoters and genes in order to identify candidate SNP markers of diseases. Considering that quite a lot of research that includes this kind of annotation has already been carried out, we present one of the options as an example

This article presents the results of a genome-wide data analysis, including features of the distribution of genes by the number of transcripts, the distribution of SNPs affecting the affinity of TBP for DNA by positions within promoters. The article also presents patterns connecting the affinity of TBP for the promoter, the specificity of the binding site of TBP for the promoter, and other characteristics of promoters that are important for the functional classification of promoters and the study of features of differential gene expression.

## Materials and methods

**Fig. 1.**
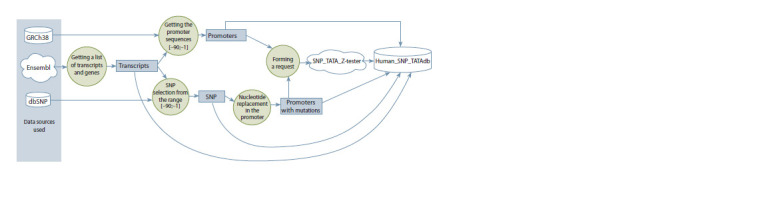
Data flow diagram for initializing the Human_SNP_TATAdb database.

Below, we present a data flow diagram for data integration and database initialization (Fig. 1). Further, all stages of work are described in more detail. Data on genes and their attributes, transcription starts and transcripts were obtained from the Ensembl web service (Birney et al., 2004). To access the services and databases used in the work, the Bioconductor library of the R language was used, with the following packages:

1. biomaRt https://bioconductor.org/packages/release/bioc/html/biomaRt.htmlis a package that provides an interface to the ENSEMBL collection of databases, allowing large volumes of data to be retrieved in a unified way and used in data analysis in Bioconductor

2. BSgenome.Hsapiens.NCBI.GRCh38 https://bioconductor.org/packages/release/data/annotation/html/BSgenome.Hsapiens.NCBI.GRCh38.html is a package that provides access to the Homo sapiens (Human) genome sequence provided by NCBI (GRCh38.p13).

3. SNPlocs.Hsapiens.dbSNP155.GRCh38https://bioconductor.org/packages/release/data/annotation/html/SNPlocs.Hsapiens.dbSNP155.GRCh38.html is a dbSNP 155 access package including information on 949,021,448 SNPs in chromosomes 1–22, X, Y and MT that was extracted from RefSNP JSON files.

To identify the start of transcription, it is necessary to use transcripts with high-quality annotation that includes this in- formation and for which there is evidence of their biological relevance. When annotating transcripts in Ensembl, special tags identify the highest quality annotated transcripts. We included in the database only those transcripts, the annota- tion quality of which corresponds to the “GENCODE Basic” label https://www.ensembl.org/info/genome/genebuild/transcript_quality_tags.html. According to the specification, the Enseml GENCODE Basic set contains at least one transcript for each gene in the GENCODE genetic set, regardless of biotype, i.e. each gene is represented in the core GENCODE set. For protein-coding genes, only full-length protein-coding transcripts are included in the core GENCODE set

For the specified transcription start coordinates, the coordi- nates and nucleotide sequences of the corresponding promoter are determined ([–90; –1] from the transcription start). We obtained SNP data using the dbSNP database https://www.ncbi.nlm.nih.gov/snp/ (Sherry et al., 2001). For each promoter, SNPs located within [–90; –1] from the start of transcription were identified. Minor promoter sequence variants were created automatically by adding cor- responding nucleotide substitutions from the dbSNP database (issue 155) to the major sequence variants.

The Bucher weight matrix (Bucher, 1990) was used to identify TATA-containing promoters.

The affinity of TBP for DNA was calculated using a three- step binding model previously developed at the Institute of Cytology and Genetics SB RAS (Ponomarenko et al., 2008) and a multi-threaded high-performance version of the SNP_TATA_Z-tester program also implemented by us. This program also allows you to evaluate the statistical significance of changes in the affinity of the TBP protein for the promoter due to point nucleotide substitutions (SNPs) in the promoter using a z-test

The affinity of TBP is described by the association constant of the TBP/DNA complex. However, at present, instead of the association constant, the inverse measure is usually used – the dissociation constant Kd. In this case, the affinity of TBP for DNA, measured in nanomoles per liter (nM/L), will be equal to А = 109/Kd. The lower the Kd, the higher the affinity of TBP for the promoter and the stronger the interaction of TBP with the promoter

The second option presented in the database is the logarith- mic form of affinity α = 9*ln(10) – ln(Kd), which is convenient for comparing TBP affinity to the promoter, since it has a distribution close to normal. As α increases, the affinity of TBP for the promoter and the strength of their interaction increase.

We performed affinity calculations for reference DNA sequences of all promoters and minor sequence variants of these promoters with one single nucleotide polymorphism. For each minor sequence, we assessed the deviation of TBP affinity for the promoter from the affinity obtained for the promoter DNA sequence from the reference genome. At the same time, the level of statistical significance of these changes was determined

It was previously shown that the affinity of TBP for the promoter is statistically significantly correlated with the level of expression of the corresponding transcript (Mogno et al., 2010). Therefore, with a statistically significant increase or decrease in TBP affinity, an estimate of the corresponding change in the level of transcript expression is indicated in the database.

Based on the estimates of TBP protein-promoter affinity, we introduced additional characteristics, such as TBP protein-promoter binding site specificity, which are useful for promoter classification and biological annotation of groups of promoters or genes.

The specificity of the binding site of TBP for the gene promoter corresponds to the maximum normalized affinity of TBP for the gene promoter relative to the average affinity of TBP for each position of the sliding window (Ponomarenko et al., 2015), not including 10 positions closest to the start of transcription (55 values in total). Specificity Z was calculated as follows:

**Formula. 1.**
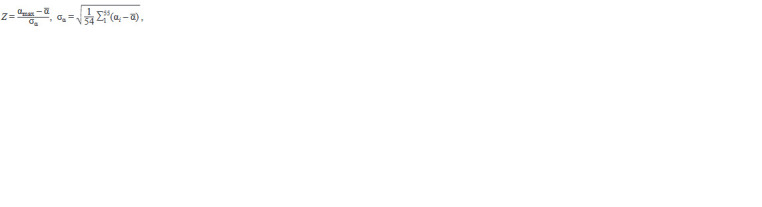
Formula. 1.

where αi is the assessment of the affinity of TBP to the pro- moter at position i, α is the average value of αi, σα is the unbiased estimate of standard deviation αi, Z is the specificity of the TBP protein binding site for the promoter.

Another important indicator describing the change in the affinity of TBP for the promoter caused by SNP is the natural logarithm of the Kd ratio for the reference (wt) and minor (mt) alleles of the SNP in question, which is calculated as follows

**Formula. 2.**
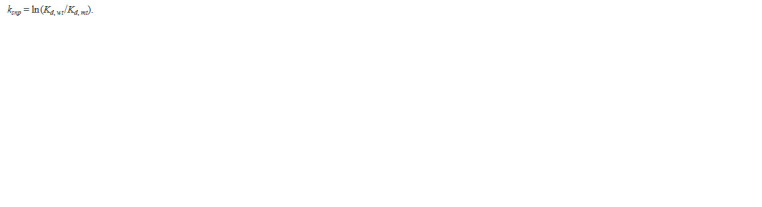
Formula. 2.

## Results and discussion

Database

**Fig. 2.**
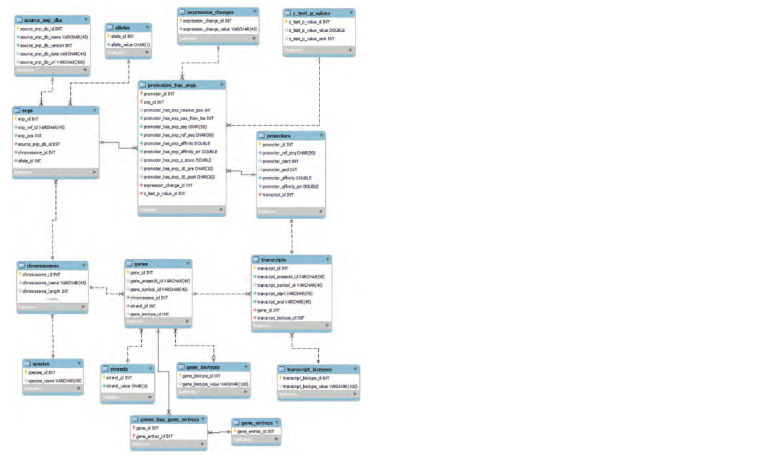
Scheme of the Human_SNP_TATAdb database.

The Human_SNP_TATAdb database has been developed, its logical diagram is presented in Fig. 2. The database was populated in accordance with the data integration and database initialization scenario presented in Fig. 1

The database is implemented on the basis of the MySQL DBMS https://www.mysql.com/ version 8.0 and includes 6 main tables (chromosomes, genes, transcripts, snps, promoters, promoters_has_snps), 10 uxiliary tables and dictionaries. The diagram of the developed database is shown in Fig. 2. Queries to the database are carried out through SQL queries.

The chromosomes table includes the chromosome identi- fier, length, number of nucleotides, and species of organism

The genes table includes information about gene identifiers in various databases, including ensembl, gene symbol name, chromosome reference, chain, and gene biotype.

The transcripts table includes information about transcript identifiers, transcript coordinates in the genome, transcript biotype, and a link to the promoter and the gene.

The snps table includes the following information: SNP identifiers, SNP positions in the genome, chromosome re- ference and allele. Here and below, one SNP is taken to be an unambiguous variant of a genome change. Polymorphisms that have one rs identifier, but allow several nucleotide substitution options, are counted by the number of such options

It should be noted that the same nucleotide substitution can occur in different gene promoters and differently change the level of affinity of the TBP protein for these promoters, and therefore two tables are defined in the database to de - scribe the promoters, promoters and promoters_has_snps, with a 1:N ratio (one promoter can influence several SNPs), and the snps and promoters_has_snps tables are also related by a 1:N relationship (one SNP can be included in several promoters).

The first promoters table includes the following informa- tion: promoter identifier, DNA sequence corresponding to the region [–90; –1] from the start of transcription, coordinates of the start and end of the promoter in the genome, affinity of the TBP protein for the promoter with an error, link to the gene.

The promoters_has_snps table includes information about the promoter identifier, a link to SNP, coordinates of SNP in the promoter and relative to the start of transcription, the sequence of the wild-type promoter and the promoter with SNP, the affinity of TBP for the promoter with an error, the nature of changes in gene expression due to mutation in the promoter, the significance level of the statistical test.

The source_snp_dbs table includes information about data sources, database versions, links to databases, which is necessary for automated updating of the Human_SNP_ TATAdb database.

Table relationship types define constraints that match the provenance of the data and are therefore important for maintaining the integrity of the database, as well as for providing additional control over the data and reducing the possibility of errors. In particular, each gene may have one or more promoters, and each promoter may regulate the expression of one or more transcripts.

As a result, the database contains the following information: • 62603 genes, of which 19314 encode proteins. • 117414 transcripts, of which 63141 encode proteins. • 5,305,816 SNP variants in gene promoters in the [–90; –1] interval from the start of transcription, of which 3,199,285 are in the promoters of protein-coding genes. • For 445,875 SNP variants in the promoter of a protein- coding gene, we predicted that they statistically significantly (p-value < 0.05) change the level of TBP affinity for this promoter.

Application options for the Human_SNP_TATAdb database

The information presented in the database (affinity of the TBP protein for the promoter, specificity of the binding site of TBP for the promoter and assessment of changes in these characteristics due to SNP) may be important for identification of markers of genetic susceptibility to diseases, identification and functional interpretation of classes of promoters similar in the mechanism of regulation of the early stage transcription initiation, etc.

The Human_SNP_TATAdb database can also help to an- notate genes or a group of genes in terms of TBP affinity for a promoter or TBP binding site specificity for a promoter. To determine the characteristic of a gene associated with the specific binding of TBP to gene promoters for the purpose of GO analysis, you can use the average values of the affinity of TBP for gene promoters or the affinity of TBP for the promoter corresponding to the only transcript for the gene, which is determined by ENSEMBL experts as canonical and is speci- fied in the database with the label “Ensembl Canonical” https://www.ensembl.org/info/genome/genebuild/canonical.html, i.e. it is generally the most conserved, the most expressed, has the longest coding sequence, and is represented in other key resources such as NCBI and UniProt. We mark its correspond- ing promoter as canonical and use characteristics such as the affinity of TBP for the canonical promoter and the specificity of the TBP binding site for the canonical promoter to annotate a gene or group of genes.

Correlation analysis showed that there is a strong linear relationship between the affinity of TBP for the canonical gene promoter and the average affinity of gene promoters (R = 0.88, d.f. = 19308). Therefore, using any option will lead to similar results. However, using TBP’s affinity for the ca- nonical gene promoter appears to be biologically more reason- able. Of course, a key use case for the Human_SNP_TATAdb database is gene annotation and identification of candidate SNP markers for disease susceptibility

Considering that to date quite a lot of studies have already been conducted in which this kind of annotation has been car- ried out, we will present the work (Bogomolov et al., 2023) as an example using the Human_SNP_TATAdb database for annotation and identification of candidate SNP markers of atherogenesis, atherosclerosis and atheroprotection

We pre-selected 1068 human genes associated with these diseases. Information about single nucleotide polymorphisms in the promoters of these human genes, the results of assessing the affinity of TBP for promoters and assessing their effect on gene expression for wild-type promoters and promoters with SNP was obtained from the Human_SNP_TATAdb database. This information was supplemented by an annotation of se- lected genes prepared by experts, and a database view was generated, focused on the analysis of genes associated with atherogenesis, atherosclerosis and atheroprotection, external access to which is provided via the Web interface http://www.sysbio.ru/Human_SNP_TATAdb

In silico analysis of all 5112 SNPs in their promoters identi- fied 330 candidate SNP markers that statistically significantly alter the affinity of TBP for these promoters.

Next, we compared the corresponding frequencies of SNPs that increase and decrease the affinity of TBP for the promoters of the same genes. This comparison was made to analyze whether these genes are under the influence of natu- ral selection or neutral drift. We found that natural selection acts against underexpression of hub genes for atherogenesis, atherosclerosis and atheroprotection and, through enhanced atheroprotection, contributes to improved human health (Bo- gomolov et al., 2023).

Examples of application of the Human_SNP_TATAdb database for genome-wide analysis

The developed database makes it possible to analyze genome- wide statistics and the distribution of these indicators in various groups of promoters, for example, TATA-containing promo- ters. For genome-wide analysis, we used protein-coding genes and transcripts selected by the values of the ‘gene_biotype’ and ‘transcript_biotype’ fields equal to ‘protein_coding’.

Alternative promoters and TBP/DNA affinity

**Fig. 3.**
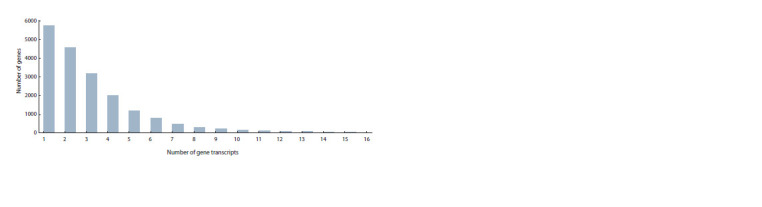
Distribution of protein-coding genes by number of transcripts.

It should be noted that one gene can have several transcripts, the initiation of transcription of which occurs using different promoters, for which the affinity of the TBP protein is assessed. Figure 3 shows the distribution of protein-coding genes by transcript number. The largest number of protein- coding genes (29.77% of genes) have a single transcript and, as a consequence, one promoter. 5% of protein-coding genes have at least 9 protein-coding transcripts. Analysis of the distribution of genes by the number of transcripts showed that the average number of transcripts per gene is 3.27, and the median is 2 transcripts per gene. The Mapk10 (mitogen- activated protein kinase 10) gene has the maximum number of protein-coding transcripts (87).

Our analysis showed that the distribution of the average affinity of TBP for canonical promoters in groups of genes divided by the number of transcripts is close to uniform. Thus, there is no need to neutralize the effects due to different numbers of transcripts per gene when analyzing data using TBP affinity.

Distribution of SNPs that change gene expression by promoter positions

**Fig. 4.**
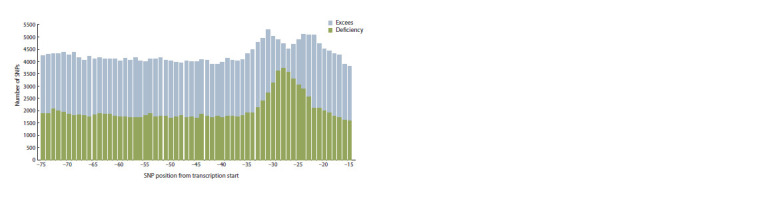
Distribution of the number of SNPs that increase (excess) and decrease (deficiency) the affinity of TBP for the DNA of the promoters of protein-coding genes, depending on the position of the SNP relative to the start of transcription

The distribution of SNPs that statistically significantly change gene expression at positions from the start of transcription is clearly different from uniform (Fig. 4). In the region [–35; –20], corresponding to the usual location of the TATA box, the number of such SNPs is noticeably higher than in other regions of the promoter. The number of SNPs that reduce gene expression in the [–35; –20] region, corresponding to the location of the TATA-box, is more than one and a half higher than in other regions of the promoter. This may be due to the fact that SNPs in this region tend to disrupt the TATA box

The number of SNPs that increase gene expression is higher on the flanks of the most frequent TATA box locations. The peaks are located at positions –24 and –32 from the start of transcription. It should be noted that the distribution of all SNPs across the promoter positions of protein-coding genes is uniform. This suggests that an increase in the number of SNPs that increase gene expression on the flanks of the TATA box may have functional significance.

Affinity of TBP to TATA-containing and TATA-free promoters of protein-coding genes

**Fig. 5.**
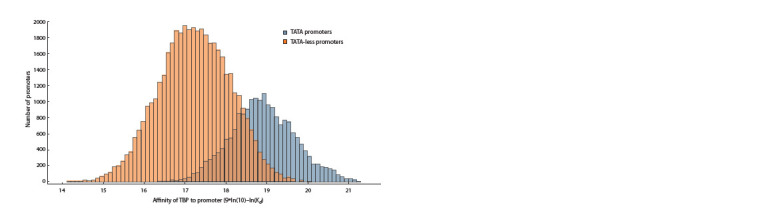
Distribution of promoters of protein-coding genes by TBP affinity in groups of TATA-containing promoters and promoters without a TATA box. The x-axis TBP affinity score for the promoter is given on a logarithmic scale.

Analysis of the dependence of TBP/DNA affinity indicators, measured on a logarithmic scale (α = 9*ln(10) – – ln(Kd), for TATA-containing and TATA-free promoters of protein-coding genes (Fig. 5), showed that the group of TATA-containing promoters exhibits higher TBP/DNA affinity, consistent with stronger TBP-promoter affinity

Functional SNPs affecting the affinity of TBP for promoter DNA and the specificity of the TBP protein binding site

**Fig. 6.**
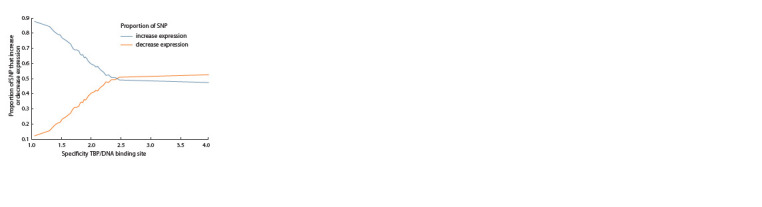
Proportion of SNPs in promoters that increase and decrease the expression of protein-coding genes depending on the specificity of the TBP binding site for the DNA promoter.

We analyzed the dependence of the proportion of SNPs that have a statistically significant effect on the affinity of TBP for the DNA of the promoters of protein-coding genes on the specificity of the TBP protein binding site (Fig. 6). It has been shown that SNPs in promoters with low specificity of the TBP binding site for the promoter, as a rule, lead to an increase in gene expression, and in promoters with high specificity, the proportion of SNPs that decrease expression is increased

Analysis of the contingency table showed that low specificity values of the TBP binding site to the promoter (spec less than 2.5) are more often observed in promoters without a TATA box (TATA–) (χ2 = 10 385, p-value < 1.0e–228)

**Table. 1.**
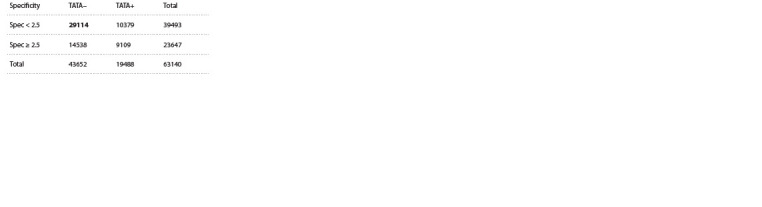
Contingency table of the specificity of the TBP binding site with the promoter and the presence of a TATA box in the promoter

## Conclusion

This work presents the Human_SNP_TATAdb database, which includes information on single nucleotide polymorphisms in human gene promoters obtained by automatic extraction from various heterogeneous data sources, the results of assessing the affinity of TBP for the promoter using a three-step binding model, and assessing their impact on gene expression for wild-type promoters and promoters with a single nucleotide polymorphism

The affinity of the TBP protein for the promoter, the specificity of the TBP binding site for the promoter, and assessments of changes in these characteristics with single nucleotide polymorphisms presented in the database may be important for identification of candidate markers of genetic susceptibility to diseases, identification and functional interpretation of classes of promoters that are similar in the mechanism of regulation of the early stage of transcription initiation, etc.

The Human_SNP_TATAdb database can also be used to annotate genes or groups of genes in terms of TBP-promoter affinity or TBP-promoter binding site specificity.

The results of genome-wide analysis showed that the affinity of TBP for the promoter and the specificity of its binding site are statistically associated with other characteristics of promoters that are important for the functional classification of promoters and the study of differential gene expression patterns.

The use of the Human_SNP_TATAdb database for gene annotation and the identification of candidate SNP markers of atherogenesis, atherosclerosis and atheroprotection is one example, as a result of which new knowledge is emerging about the effect of various single polymorphisms on susceptibility to certain diseases.

## Conflict of interest

The authors declare no conflict of interest.

## References

Birney E., Andrews T.D., Bevan P., Caccamo M., Chen Y., Clarke L.,
Coates G., ..., Cox A., Hubbard T., Clamp M. An overview of En-
sembl. Genome Res. 2004;14(5):925-928. DOI 10.1101/gr.1860604

Bogomolov A., Filonov S., Chadaeva I., Rasskazov D., Khandaev B.,
Zolotareva K., Kazachek A., … Kolchanov N., Tverdokhleb N.,
Ponomarenko M. Candidate SNP markers significantly altering the affinity of TATA-binding protein for the promoters of human hub
genes for atherogenesis, atherosclerosis and atheroprotection. Int. J.
Mol. Sci. 2023;24(10):9010. DOI 10.3390/ijms24109010

Bucher P. Weight matrix descriptions of four eukaryotic RNA poly-
merase II promoter elements derived from 502 unrelated promoter
sequences. J. Mol. Biol. 1990;212(4):563-578. DOI 10.1016/0022-
2836(90)90223-9

Chadaeva I.V., Ponomarenko M.P., Rasskazov D.A., Sharypova E.B.,
Kashina E.V., Matveeva M.Yu., Arshinova T.V., Ponomarenko P.M.,
Arkova O.V., Bondar N.P., Savinkova L.K., Kolchanov N.A. Candi-
date SNP markers of aggressiveness-related complications and co-
morbidities of genetic diseases are predicted by a significant change
in the affinity of TATA-binding protein for human gene promoters.
BMC Genomics. 2016;17(Suppl. 14):995. DOI 10.1186/s12864-016-
3353-3

Chandra V., Bhattacharyya S., Schmiedel B.J., Madrigal A., Gonzalez-
Colin C., Fotsing S., Crinklaw A., Seumois G., Mohammadi P.,
Kronenberg M., Peters B., Ay F., Vijayanand P. Promoter interacting
expression quantitative trait loci are enriched for functional genetic
variants. Nat. Genet. 2021;53(1):110-119. DOI 10.1038/s41588-
020-00745-3

Delgadillo R.F., Whittington J.E., Parkhurst L.K., Parkhurst L.J. The
TATA-binding protein core domain in solution variably bends TATA
sequences via a three-step binding mechanism. Biochemistry. 2009;
48(8):1801-1809. DOI 10.1021/bi8018724

French J.D., Edwards S.L. The role of noncoding variants in heritable
disease. Trends Genet. 2020;36(11):880-891. DOI 10.1016/j.tig.
2020.07.004

Hindorff L.A., Sethupathy P., Junkins H.A., Manolio T.A. Potential
etiologic and functional implications of genome-wide association
loci for human diseases and traits. Proc. Natl. Acad. Sci. USA. 2009;
106(23):9362-9367. DOI 10.1073/pnas.0903103106

Maurano M.T., Humbert R., Rynes E., Thurman R.E., Haugen E.,
Wang H., Reynolds A.P., … Sunyaev S.R., Kaul R., Stamatoyanno-
poulos J.A. Systematic localization of common disease-associated
variation in regulatory DNA. Science. 2012;337(6099):1190-1195.
DOI 10.1126/science.1222794

Mogno I., Vallania F., Mitra R.D., Cohen B.A. TATA is a modular
component of synthetic promoters. Genome Res. 2010;20(10):1391-
1397. DOI 10.1101/gr.106732.110

Oshchepkov D., Chadaeva I., Kozhemyakina R., Zolotareva K., Khan-
daev B., Sharypova E., Ponomarenko P., Bogomolov A., Klimo-
va N.V., Shikhevich S., Redina O., Kolosova N.G., Nazarenko M.,
Kolchanov N.A., Markel A., Ponomarenko M. Stress reactivity, sus-
ceptibility to hypertension, and differential expression of genes in
hypertensive compared to normotensive patients. Int. J. Mol. Sci.
2022;23(5):2835. DOI 10.3390/ijms23052835

Ponomarenko P.M., Savinkova L.K., Drachkova I.A., Lysova M.V., Ar-
shinova T.V., Ponomarenko M.P., Kolchanov N.A. A step-by-step
model of TBP/TATA box binding allows predicting human heredi-
tary diseases by single nucleotide polymorphism. Dokl. Biochem.
Biophys. 2008;419:88-92. DOI 10.1134/S1607672908020117

Ponomarenko M., Rasskazov D., Arkova O., Ponomarenko P., Su-
slov V., Savinkova L., Kolchanov N. How to use SNP_TATA_Com-
parator to find a significant change in gene expression caused by
the regulatory SNP of this gene’s promoter via a change in affin-
ity of the TATA-binding protein for this promoter. Biomed Res. Int.
2015;2015:359835. DOI 10.1155/2015/359835

Ponomarenko M.P., Arkova O., Rasskazov D., Ponomarenko P., Savin-
kova L., Kolchanov N. Candidate SNP markers of genderbiased
auto immune complications of monogenic diseases are predicted by
a significant change in the affinity of TATA-binding protein for hu-
man gene promoters. Front. Immunol. 2016a;7:130. DOI 10.3389/
fimmu.2016.00130

Ponomarenko P., Rasskazov D., Suslov V., Sharypova E., Savinko-
va L., Podkolodnaya O., Podkolodny N.L., Tverdokhleb N.N., Cha-
daeva I., Ponomarenko M., Kolchanov N. Candidate SNP markers
of chronopathologies are predicted by a significant change in the af-
finity of TATA-binding protein for human gene promoters. Biomed
Res. Int. 2016б;2016:8642703. DOI 10.1155/2016/8642703

Ponomarenko M., Rasskazov D., Chadaeva I., Sharypova E., Ponoma-
renko P., Arkova O., Kashina E., Ivanisenko N., Zhechev D., Sa-
vinkova L., Kolchanov N. SNP_TATA_Comparator: genomewide
landmarks for preventive personalized medicine. Front. Biosci.
(Schol. Ed.). 2017;9(2):276-306. DOI 10.2741/s488

Rasskazov D.A., Gunbin K.V., Ponomarenko P.M., Vishnevsky O.V.,
Ponomarenko M.P., Afonnikov D.A. SNP_TATA_COMPARATOR:
web service for comparison of SNPS within gene promotеrs asso-
ciated with human diseases using the equilibrium equation of the
TBP/TATA complex. Vavilovskii Zhurnal Genetiki i Selektsii = Vavi-
lov Journal of Genetics and Breeding. 2013;17(4/1):599-606 (in
Russian)

Savinkova L.K., Drachkova I.A., Ponomarenko M.P., Lysova M.V.,
Arshinova T.V., Kolchanov N.A. Interaction of recombinant TATA-
binding protein with mammals gene promoter TATA boxes. Eko-
logicheskaya genetika = Ecological genetics. 2007;5(2):44-49. DOI
10.17816/ecogen5244-49 (in Russian)

Savinkova L., Drachkova I., Arshinova T., Ponomarenko P., Ponoma-
renko M., Kolchanov N. An experimental verification of the pre-
dicted effects of promoter TATA-box polymorphisms associated
with human diseases on interactions between the TATA boxes and
TATA-binding protein. PLoS One. 2013;8(2).e54626. DOI 10.1371/
journal.pone.0054626

Sherry S.T., Ward M.H., Kholodov M., Baker J., Phan L., Smigiel-
ski E.M., Sirotkin K. dbSNP: the NCBI database of genetic varia-
tion. Nucleic Acids Res. 2001;29(1):308-311. DOI 10.1093/nar/29.
1.308

